# HPV prevalence among young adult women living with and without HIV in Botswana for future HPV vaccine impact monitoring

**DOI:** 10.1186/s12879-022-07130-x

**Published:** 2022-02-22

**Authors:** Nancy McClung, Anikie Mathoma, Julia W. Gargano, Naledi Gape Nyepetsi, Troy D. Querec, Juanita Onyekwuluje, Madisa Mine, Chelsea Morroni, Rebecca Luckett, Lauri E. Markowitz, Doreen Ramogola-Masire

**Affiliations:** 1 National Center for Immunization and Respiratory Diseases, Centers for Disease Control and Prevention, 1600 Clifton Rd NE, Atlanta, GA 30329 USA; 2 Faculty of Medicine, Office of Research and Graduate Studies, University of Botswana, Plot 4775 Notwane Rd., Gaborone, Botswana; 3 National Center for Emerging, Zoonotic, and Infectious Diseases, Centers for Disease Control and Prevention, 1600 Clifton Rd NE, Atlanta, GA 30329 USA; 4 National Health Laboratory, Botswana Ministry of Health and Wellness, Plot 5353 Church Road, Extension 10, Gaborone, Botswana; 5 MRC Centre for Reproductive Health, University of Edinburgh, Old College, South Bridge, Edinburgh, EH8 9YL UK; 6 Department of Obstetrics and Gynecology, Beth Israel Deaconess Medical Center, 330 Brookline Ave, Boston, MA 02215 USA; 7 Department of Obstetrics and Gynecology, Faculty of Medicine, University of Botswana, Plot 4775 Notwane Rd., Gaborone, Botswana

**Keywords:** Human papillomavirus, Human immunodeficiency virus, HPV vaccine impact, Perinatal HIV infection

## Abstract

**Introduction:**

In 2015, Botswana introduced quadrivalent human papillomavirus (HPV) vaccine for girls aged 9–13 years. To establish a baseline HPV prevalence for future HPV vaccine impact monitoring, we evaluated HPV prevalences among the youngest unvaccinated women in Botswana and compared HPV prevalences among women living with HIV (WLHIV) and without HIV.

**Methods:**

Women aged 18–22 years were recruited from the University of Botswana and HIV clinics in Gaborone from October 2019–January 2021. Demographic and behavioral characteristics were self-reported during structured interviews; HIV clinical characteristics were abstracted from medical charts. Self-collected vaginal swabs were tested for 28 HPV types using Seegene Anyplex II HPV28. We compared prevalence of any HPV, high risk (HR)-HPV, and quadrivalent HPV vaccine types (HPV6/11/16/18) among WLHIV and women without HIV and evaluated risk factors for prevalence of HR-HPV.

**Results:**

A total of 306 WLHIV and 500 women without HIV were recruited. Compared to women without HIV, WLHIV were more likely to be sexually experienced (86.6% versus 74.4%) and have ≥ 3 lifetime sex partners (55.3% versus 27.8%). All HPV type prevalences were significantly higher among WLHIV compared to women without HIV, including prevalence of any HPV (82.7% versus 63.0%), HR-HPV (72.9% versus 53.8%), and quadrivalent vaccine HPV types (34.3% versus 21.0%). Among WLHIV, there were no differences between those perinatally and non-perinatally infected for HPV prevalences, number of HPV types detected, CD4 count, or viral load.

**Conclusions:**

Over one-third of WLHIV and nearly a quarter of those without HIV had vaccine-type HPV detected. This study supports need for the national HPV vaccination program in Botswana and provides important baseline data for future evaluation of impact of the program.

## Background

Cervical cancer is the most common cancer among women in Botswana and elsewhere in Sub-Saharan Africa [1]. Human papillomavirus (HPV) infection causes cervical cancer and other anogenital and oropharyngeal cancers [2]. HPV types 16 and 18 are associated with ~ 70% of cervical cancer worldwide [3]. Women living with HIV (WLHIV) have almost five times the risk of cervical cancer compared to women without HIV [4]. Botswana has a high prevalence of HIV [5], with approximately 28% of women aged 15–49 years living with HIV [6].

In 2015, Botswana introduced HPV vaccine into their national immunization program for the prevention of cervical and other HPV-associated cancers [1, 7]. Quadrivalent HPV vaccine (which targets HPV types 6, 11, 16, and 18) is delivered through school-based programs in a 2-dose schedule for girls without HIV and a 3-dose schedule for girls living with HIV, in accordance with World Health Organization (WHO) recommendations [8]. In the first year of the immunization program, girls aged 9–13 years were vaccinated, followed by girls aged 9 years in subsequent years. Over 90% 2-dose coverage of girls in the targeted age range was achieved in the first year of the program (personal communication, Botswana Ministry of Health and Wellness). Botswana was the second country in Africa to introduce HPV vaccination into their national program and the first country in Africa to introduce a 2-dose vaccination schedule [9].

Impact of HPV vaccination programs in many high-income countries has been observed through the reduction in prevalence of HPV vaccine targeted types and other HPV attributable outcomes [10]. Little information, however, is available on the impact of HPV vaccination programs in low- and middle-income countries. Further, among WLHIV, there are few data on efficacy or effectiveness of HPV vaccine, although immunogenicity studies show that most persons living with HIV develop antibodies after vaccination [11, 12]. The aims of this study were to: (1) establish baseline HPV vaccine and non-vaccine-type prevalences among WLHIV aged 18–22 years in Botswana for future evaluation of HPV vaccine impact, (2) compare HPV prevalences between WLHIV and women living without HIV, and (3) to determine risk factors for high-risk (HR)-HPV infection.

## Methods

We analyzed data from female participants in the Baseline HPV Prevalence for Vaccine Impact Monitoring in Botswana Study, a cross-sectional study of HPV prevalence among young adults aged 18–22 years. Although HPV vaccine was introduced in the national immunization program in Botswana in 2015, a baseline HPV prevalence could still be established among young adult women because girls vaccinated in the first year of the national program were 13–17 years-old in 2019, when the study recruitment began. Participants were consecutively recruited from the University of Botswana and HIV clinics in Gaborone, Botswana, from October 2019–January 2021, with the majority of participants recruited by July 2020. The inclusion criteria were documented HIV status in the prior 12 months (Ministry of Health HIV status card or rapid HIV test at enrollment) and age 18–22 years. Exclusion criteria included unknown HIV status, refusal of access to medical records to obtain information on HIV treatment and care, or known current pregnancy. Informed consent was provided by all participants prior to any data collection. Participants self-reported demographic and behavioral characteristics during structured interviews and self-collected a vaginal swab. Clinical data were abstracted from medical records for WLHIV.

DNA was isolated from specimens with proteinase K lysis followed by automated extraction in the NucliSENS easyMAG (bioMérieux SA, Marcy-l'Étoile, France) following manufacturer’s instructions. Swabs in the specimen transport medium (STM) were agitated on an orbital shaker for 45 min at ambient room temperature. For specimen lysis, 200 µL of vaginal swab STM was incubated with 20 µL proteinase K (catalog number CMG-1077, Perkin Elmer, Shelton, CT, USA) and enough NucliSENS easyMAG Lysis Buffer (catalog # 280134, bioMérieux) to bring the total processing volume to 800 µL. After incubation at 65 °C for 1 h, extraction was completed on NucliSENS EasyMAG (bioMérieux). DNA was eluted in 100 µL of buffer 3 at a pH > 8.0 and temperature of 70 °C. Water aliquots were processed in parallel with specimens as negative controls for DNA contamination.

Extracts were tested according to manufacturer’s End Point-CMTA protocol with Anyplex II HPV28 assay (catalog # HP7S00X; Seegene, Seoul, Korea). This assay detects 28 HPV types (6, 11, 16, 18, 26, 31, 33, 35, 39, 40, 42, 43, 44, 45, 51, 52, 53, 54, 56, 58, 59, 61, 66, 68, 69, 70, 73, and 82) and human internal control DNA. The assay was conducted on CFX96 Dx System (catalog #IBRD1855196, Bio-Rad, Hercules, California, USA). Automated analysis by Seegene Viewer software interpreted results as HPV positive, HPV negative or invalid.

This analysis was restricted to participants with valid HPV typing results. Demographic variables included age, sex, marital status (single versus married/living together), and education (vocational college or more, secondary, part of secondary or less). Behavioral characteristics included ever having sex (yes, no), number of sex partners in past 12 months (0, 1, 2, ≥ 3), lifetime number of sex partners (0, 1, 2, 3–5, ≥ 6), age of first sex (vaginal, oral, or anal sex, < 18, ≥ 18 years), age of first sex partner (< 20 versus ≥ 20 years), age of current sex partner (< 24, ≥ 24 years), sexual orientation (heterosexual/straight, homosexual/gay or lesbian, bisexual, other), condom use in past 3 months (always, sometimes/never, no sex in past 3 months), tobacco use (daily, weekly, none), and ever pregnant (yes, no). HIV status (positive, negative) was based on documentation obtained at enrollment. Among WLHIV, the most recent CD4 count (< 200, 200–349, 350–499, ≥ 500 cells/mm^3^), most recent viral load (< 400, ≥ 400 copies/mL), and age of antiretroviral therapy (ART) initiation (0–10, 11–17, ≥ 18 years) were abstracted from medical charts. Perinatal infection was ascertained via self-report (yes, no, unknown). A small number of participants (n = 18) reported perinatal infection status that appeared implausible in light of other self-reported or chart-verified information and were reclassified accordingly: participants who started ART at age < 15 years but self-reported non-perinatal or unknown infection were re-classified as perinatally infected (n = 6). Participants who started ART at age ≥ 15 years and within 1 year of first sex, and self-reported perinatal infection, were re-classified as not perinatally infected (n = 12). Although study participants were not expected to have received HPV vaccination based on age eligibility, self-reported HPV vaccination status was obtained to identify any bias in baseline HPV prevalence results. Only 4% of study participants reported plausible HPV vaccination based on age and geographic location; due to the small number, these participants were included in the analysis.

HPV types were categorized as any HPV, any HR-HPV (HPV16, 18, 31, 33, 35, 39, 45, 51, 52, 56, 58, 59, 66, 68), any low-risk (LR)-HPV (HPV6, 11, 26, 40, 42, 43, 44, 53, 54, 61, 69, 70, 73, 82), quadrivalent vaccine types (HPV6, 11, 16, 18), HR-HPV vaccine types (HPV16, 18), and LR-HPV vaccine types (HPV6, 11). Among participants with any HPV detected, number of HPV types detected were categorized as 1, 2–4, and ≥ 5.

Demographic and behavioral characteristics were described by HIV status. HPV prevalences were also described by HIV status for both individual HPV types and HPV type categories, and among sexually experienced participants for HPV type categories. HPV prevalences and HIV clinical characteristics were also described by perinatal HIV infection status among sexually experienced WLHIV.

Because HR-HPV infections can result in more rapid neoplastic changes in WLHIV, we were interested in identifying characteristics associated with HR-HPV prevalence by HIV status. Among sexually experienced participants, unadjusted prevalence ratios (PR) and 95% confidence intervals (CI) were calculated using log-binomial regression to evaluate associations between selected participant characteristics and HR-HPV. Multivariable models were developed using backward selection (p < 0.3 to enter, p < 0.15 to stay) to identify characteristics associated with having a prevalent HR-HPV infection in this population. For the models, lifetime number of sex partners were categorized as 1–2 versus ≥ 3, CD4 count as ≤ 350 versus > 350 cells/mm^3^, and age of ART initiation as < 15 versus ≥ 15 years. A sensitivity analysis excluding perinatally-infected WLHIV was conducted, but no meaningful differences in model findings were observed (data not shown). All analyses were conducted using SAS 9.4 (Statistical Analysis Software, Cary, NC, USA).

## Results

Among 806 female participants, 306 were HIV-infected and 500 were HIV-uninfected; all participants had valid HPV typing results. Compared to women without HIV, WLHIV were more likely to be sexually experienced (86.6% versus 74.4%), have ≥ 3 sex partners in the past 12 months (20.0% versus 9.7%) and in their lifetime (55.3% versus 27.8%), and have a current sex partner ≥ 24 years-old (71.7% versus 33.2%) (Table 1). Among WLHIV, 94.7% had a viral load ≤ 400 copies/mL, 82.3% had a CD4 count ≥ 350 cells/mm^3^, and 33.7% were perinatally infected; 99.7% of WLHIV were on ART at the time of study enrollment.

**Table 1:** Participant demographic, behavioral, and clinical characteristics by HIV status

	HIV-positive N = 306 n (%)	HIV-negative N = 500 n (%)	p-value**
Age (years)			< 0.001
18	22 (7.2)	83 (16.6)	
19	40 (13.1)	182 (36.4)	
20	55 (18.0)	97 (19.4)	
21	93 (30.4)	91 (18.2)	
22	96 (31.4)	47 (9.4)	
Marital status			< 0.001
Married/living together	20 (6.5)	1 (0.2)	
Single	286 (93.5)	499 (99.8)	
Education			< 0.001
Part of secondary or less	100 (32.7)	0 (0.0)	
Secondary	120 (39.2)	0 (0.0)	
Vocational college or more	86 (28.1)	500 (100.0)	
Ever had sex			< 0.001
Yes	265 (86.6)	372 (74.4)	
No	41 (13.4)	128 (25.6)	
Number of sex partners, past 12 months^a^			0.001
0	0 (0.0)	1 (0.3)	
1	149 (56.2)	230 (61.8)	
2	63 (23.8)	105 (28.2)	
≥ 3	53 (20.0)	36 (9.7)	
Lifetime number of sex partners^a^			< 0.001
0	41 (13.4)	128 (25.6)	
1	44 (14.4)	128 (25.6)	
2	52 (17.0)	105 (21.0)	
3–5	126 (41.2)	103 (20.6)	
≥ 6	43 (14.1)	36 (7.2)	
Age of first sex (years)^a^			0.16
≥ 18	207 (78.1)	307 (82.5)	
< 18	58 (21.9)	65 (17.5)	
Age of first sex partner (years)^a^			0.002
≥ 20	199 (75.1)	235 (63.3)	
< 20	66 (24.9)	136 (36.7)	
Age of current sex partner (years)^a^			< 0.001
≥ 24	190 (71.7)	123 (33.2)	
< 24	75 (28.3)	248 (66.9)	
Sexual orientation			0.15
Heterosexual/straight	300 (98.0)	482 (96.4)	
Homosexual/gay or lesbian	2 (0.7)	1 (0.2)	
Bisexual	4 (1.3)	16 (3.2)	
Other	0 (0.0)	1 (0.2)	
Condom use in past 3 months^a^			< 0.001
Always	155 (58.5)	167 (44.9)	
Sometimes/never	88 (33.2)	180 (48.4)	
No sex past 3 months	22 (8.3)	25 (6.7)	
Tobacco use			0.19
Daily	1 (0.3)	4 (0.8)	
Weekly	4 (1.3)	16 (3.2)	
Never	301 (98.4)	480 (96.0)	
Pregnant ever			< 0.001
Yes	122 (39.9)	42 (8.4)	
No	184 (60.1)	458 (91.6)	
CD4 count (cells/mm^3^)			
< 200	9 (3.0)		
200–349	45 (14.8)		
350–499	64 (21.0)		
≥ 500	187 (61.3)		
Viral load (copies/mL)			
≤ 400	283 (94.7)		
> 400	16 (5.4)		
Age of ART initiation (years)			
0–4	12 (3.9)		
5–9	47 (15.4)		
10–14	28 (9.2)		
≥ 15	219 (71.6)		
Perinatally infected			
Yes	103 (33.7)		
No	203 (66.3)		

*ART* antiretroviral therapy

^a^Among sexually experienced participants

**Chi-square or Fisher’s exact tests

HPV prevalences were significantly higher among WLHIV compared to women living without HIV, including prevalence of any HPV (82.7% versus 63.0%) and quadrivalent vaccine-type HPV (34.3% versus 21.0%) (Table 2). Among women with HPV detected, WLHIV were also more likely to have multiple HPV types. HPV prevalences were slightly higher, and remained significantly different between groups, when limiting the analysis to sexually experienced participants.

**Table 2:** HPV prevalences and number of types by HIV status, overall and among sexually experienced participants

HPV types	All participants N = 806			Sexually experienced participants N = 637		
	HIV-positive N = 306 n (%)	HIV-negative N = 500 n (%)	Chi-square p-value	HIV-positive N = 265 n (%)	HIV-negative N = 372 n (%)	Chi-square p-value
Any HPV	253 (82.7)	315 (63.0)	< 0.001	232 (87.6)	280 (75.3)	< 0.001
Any HR-HPV	223 (72.9)	269 (53.8)	< 0.001	207 (78.1)	244 (65.6)	< 0.001
Any LR-HPV	187 (61.1)	231 (46.2)	< 0.001	174 (65.7)	209 (56.2)	0.02
Quadrivalent vaccine	105 (34.3)	105 (21.0)	< 0.001	99 (37.4)	99 (26.6)	0.004
HR-HPV16/18	75 (24.5)	71 (14.2)	< 0.001	69 (26.0)	67 (18.0)	0.01
LR-HPV6/11	47 (15.4)	55 (11.0)	0.07	47 (17.7)	52 (14.0)	0.20
Number of HPV types detected (among HPV+)						
1	51 (20.2)	84 (26.7)	0.02	42 (18.1)	62 (22.1)	0.04
2–4	111 (43.9)	150 (47.6)		102 (44.0)	141 (50.4)	
≥ 5	91 (36.0)	81 (25.7)		88 (37.9)	77 (27.5)	

*HIV* human immunodeficiency virus, *HPV* human papillomavirus, *LR* low risk (HPV vaccine types 6, 11 and HPV non-vaccine types 26, 40, 42, 43, 44, 53, 54, 61, 69, 70, 73, 82, *HR* high-risk (HPV vaccine types 16, 18 and HPV non-vaccine types 31, 33, 35, 39, 45, 51, 52, 56, 58, 59, 66, 68)

Among sexually experienced WLHIV, no differences in CD4 count or viral load were observed between perinatally and non-perinatally infected women (Table 3). Most WLHIV who were perinatally infected initiated ART at ages 5–9 years, and only 10.6% initiated ART at ages < 5 years. No differences were observed in HPV prevalences (including quadrivalent vaccine types) between these two groups, and thus they were combined for subsequent analyses.

**Table 3:** HIV clinical characteristic and HPV prevalences among perinatally and non-perinatally HIV-infected, sexually experienced women

	Non-perinatally infected N = 200 n (%)	Perinatally infected N = 65 n (%)	p-value*
HIV clinical characteristics			
CD4 count (cells/mm^3^)			0.71
< 200	5 (2.5)	0 (0.0)	
200–349	32 (16.1)	9 (13.9)	
350–499	37 (18.6)	14 (21.5)	
≥ 500	125 (62.8)	42 (64.6)	
Viral load (copies/mL)			0.06
≤ 400	186 (95.9)	57 (89.1)	
> 400	8 (4.1)	7 (10.9)	
Age of ART initiation (years)			< 0.001
0–4	0 (0.0)	7 (10.8)	
5–9	0 (0.0)	34 (52.3)	
10–14	0 (0.0)	21 (32.3)	
≥ 15	199 (100.0)	3 (4.6)	
HPV type category			
Any HPV	177 (88.5)	55 (84.6)	0.41
Any HR-HPV	159 (79.5)	48 (73.9)	0.34
Any LR-HPV	132 (66.0)	42 (65.2)	0.84
Quadrivalent vaccine	72 (36.0)	27 (41.5)	0.42
HR-HPV16/18	50 (25.0)	19 (29.2)	0.50
LR-HPV6/11	33 (16.5)	14 (21.5)	0.36
Number of HPV types detected (among HPV+)			
1	30 (17.0)	12 (21.8)	0.43
2–4	76 (42.9)	26 (47.3)	
≥ 5	71 (40.1)	17 (30.9)	

*Chi-square or Fisher’s exact tests

*HIV* human immunodeficiency virus, *HPV* human papillomavirus, *LR* low risk (HPV vaccine types 6, 11 and HPV non-vaccine types 26, 40, 42, 43, 44, 53, 54, 61, 69, 70, 73, 82, *HR* high-risk (HPV vaccine types 16, 18 and HPV non-vaccine types 31, 33, 35, 39, 45, 51, 52, 56, 58, 59, 66, 68)

**Fig. 1:**
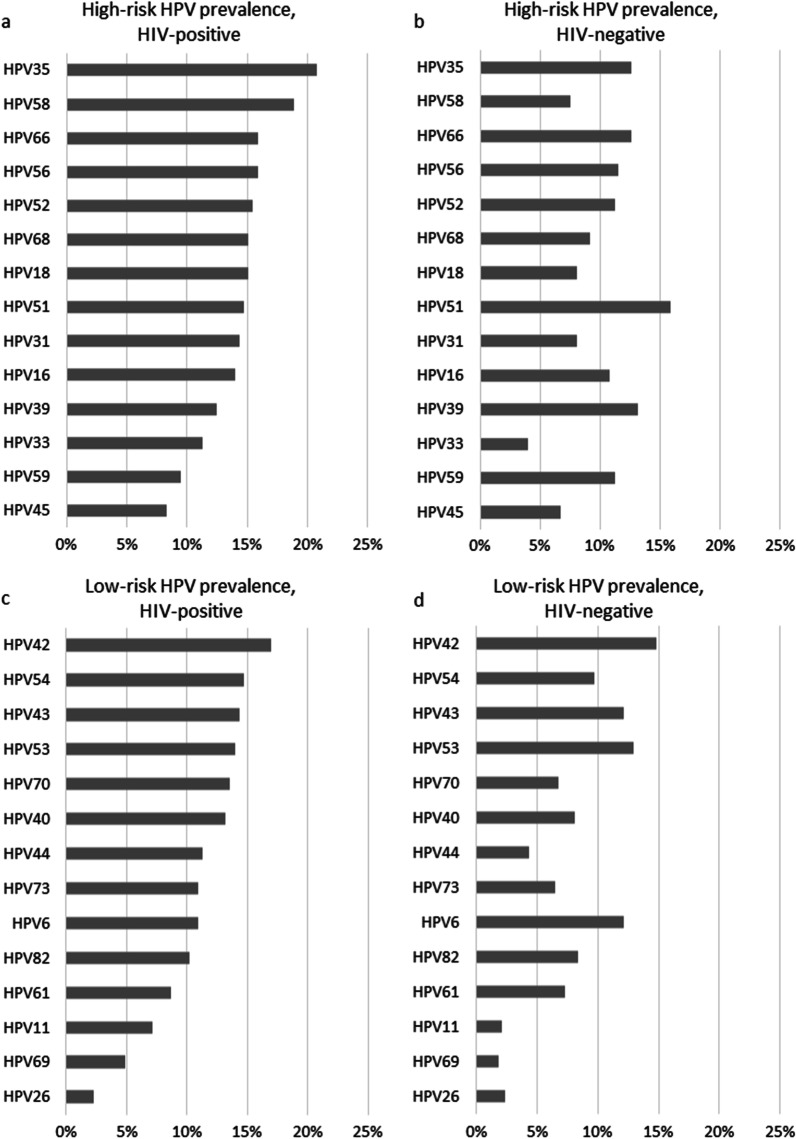
Prevalences of 28 high-risk and low-risk HPV types* among sexually experienced females by HIV status. **a** high-risk HPV prevalences, HIV-positive; **b** high-risk HPV prevalences, HIV-negative; **c** low-risk HPV prevalences, HIV-positive; **d** low-risk HPV prevalences, HIV-negative. *HPV* human papillomavirus. *HPV types ordered by magnitude among women living with HIV

The most common HR-HPV and LR-HPV types detected in WLHIV and women living without HIV are shown in Fig. 1. The five most common HR-HPV types detected among WLHIV included HPV35 (20.8%), HPV58 (18.9%), HPV66 (15.9%), HPV56 (15.9%), and HPV52 (15.5%). Among women living without HIV, the most common HR-HPV types included HPV51 (15.9%), HPV39 (13.2%), HPV66 (12.6%), HPV35 (12.6%), and HPV56 (11.6%).

Characteristics significantly associated with prevalence of any HR-HPV in unadjusted analyses included lifetime number of sex partners, ever being pregnant, and CD4 count ≤ 350 cells/mm^3^ among WLHIV; and lifetime number of sex partners and condom use in past three months among women living without HIV (Table 4). In adjusted analyses, characteristics that remained significantly associated with HR-HPV prevalence among WLHIV were CD4 count ≤ 350 cells/mm^3^ (aPR: 0.89, 95% CI 0.80, 0.99) and ever being pregnant (aPR: 0.86, 95% CI 0.76, 0.98); having ≥ 3 compared to ≤ 2 lifetime partners remained in the final model but was not statistically significant (aPR: 1.14, 95% CI 0.99, 1.32). Among women living without HIV, ≥ 3 compared to ≤ 2 lifetime partners (aPR: 1.29, 95% CI 1.12, 1.48) and always compared to sometimes or never using condoms (aPR: 0.83, 95% CI 0.71, 0.96) remained significantly associated with HR-HPV in adjusted analyses.

**Table 4:** Association between high-risk HPV prevalence and selected characteristics among sexually experienced participants, by HIV status

	HIV-positive participants N = 265			HIV-negative participants N = 372		
	High-risk HPV prevalence n (%)	Unadjusted PR (95% CI)	Adjusted^a^ PR (95% CI)	High-risk HPV prevalence n (%)	Unadjusted PR (95% CI)	Adjusted^a^ PR (95% CI)
Age of first sex (years)						
≥ 18	162 (78.3)	Ref		198 (64.5)	Ref	
< 18	45 (77.6)	1.01 (0.86, 1.18)		46 (70.8)	0.91 (0.76, 1.09)	
Age of first sex partner (years)						
≥ 20	157 (78.9)	Ref		153 (65.1)	Ref	
< 20	50 (75.8)	1.04 (0.89, 1.22)		91 (66.9)	0.97 (0.84, 1.13)	
Age of current sex partner (years)						
≥ 24	148 (77.9)	Ref		87 (70.7)	Ref	
< 24	59 (78.7)	0.99 (0.86, 1.14)		157 (63.3)	1.12 (0.96, 1.30)	
Lifetime no. sex partners						
1–2	68 (70.8)	Ref	Ref	135 (57.9)	Ref	Ref
≥ 3	139 (82.3)	1.16 (1.00, 1.34)	1.14 (0.99, 1.32)	109 (78.4)	1.35 (1.18, 1.56)	1.29 (1.12, 1.48)
Condom use in past 3 months^b^						
Sometimes/never	68 (77.3)	Ref		134 (74.4)	Ref	Ref
Always	119 (76.8)	0.99 (0.86, 1.15)		99 (59.3)	0.79 (0.68, 0.93)	0.83 (0.71, 0.96)
Ever pregnant						
No	120 (83.9)	Ref	Ref	218 (65.9)	Ref	
Yes	87 (71.3)	0.85 (0.74, .97)	0.86 (.76, .98)	26 (63.4)	0.96 (0.75, 1.23)	
CD4 count (cells/mm^3^)						
≤ 350	41 (89.1)	Ref	Ref			
> 350	165 (75.7)	0.85 (0.75, 0.96)	0.89 (0.80, 0.99)			
Viral load (copies/mL)						
> 400	15 (100.0)	–				
≤ 400	187 (77.0)	–				
ART age of initiation (years)						
< 15	46 (74.2)	Ref				
≥ 15	161 (79.3)	1.07 (0.91, 1.26)				

^a^Unadjusted prevalence ratios and 95% confidence intervals (CI) were calculated using log-binomial regression to evaluate associations between participant characteristics and any HPV. Multivariable models were developed using backward selection. (p < 0.3 to enter, p < 0.15 to stay) to identify predictors of having a prevalent HPV infection in this population

^b^Data not shown for participants reporting no sex in past 3 months

## Discussion

In this study, vaccine-type and non-vaccine-type HPV prevalences among women aged 18–22 years living with and without HIV in Botswana were characterized for the first time and established as a baseline for future evaluation of HPV vaccine impact in Botswana. HPV prevalences among WLHIV were significantly higher than women without HIV, including prevalences of HR-HPV (72.9% versus 53.8%) and quadrivalent vaccine-type HPV (34.3% versus 21.0%). There were some differences in factors associated with HR-HPV by HIV infection status. No difference in HR-HPV prevalence was observed between perinatally and non-perinatally WLHIV.

HIV infection and HIV-associated immunosuppression increase both susceptibility to and persistence of HPV infection [13]. The higher HPV prevalence among WLHIV compared to women without HIV in this report is similar to other reports among women of all ages in sub-Saharan Africa [14]. In a study of women of comparable ages in South Africa, prevalence of any HPV was very similar to this report (86.4% HIV-positive, 61.0% HIV-negative) [15], but other reports among young women found lower prevalences of HR-HPV or any HPV [16–18]. Only two other reports of quadrivalent vaccine-type HPV prevalence were identified among young women in sub-Saharan Africa. One study also found a higher prevalence among WLHIV (26% HIV-positive, 12% HIV-negative) [15]. However, both found lower prevalences than this study [15, 19]. The higher prevalence in this study may be due to geographic variability, differences in HPV specimen collection, or differences in HPV assay sensitivity.

Because some WLHIV in this study were perinatally infected, we had the ability to compare HPV prevalences in perinatally and non-perinatally infected WLHIV. Among sexually experienced WLHIV, no differences were found in HPV prevalences in perinatally infected compared to non-perinatally infected; both groups had a high prevalence of any HPV (84.9% and 88.4%, respectively). In two prior reports that directly compared perinatally and non-perinatally infected WLHIV, there were similarly no differences between the two groups in prevalence of any HPV [20, 21]. All but one woman in our study were on ART, which is associated with lower HR-HPV prevalence compared to persons who are not on ART [22, 23]. The lack of difference in viral suppression or CD4 count by perinatal infection status may also explain the similar HR-HPV prevalences. CD4 count and viral load are strongly associated with HPV infection, as observed for CD4 count in this report and other reports among WLHIV living in low- and middle-income countries [24]. Of note, only 10% of perinatally infected WLHIV in this report initiated ART before the age of 5 years, with the majority starting between the ages of 5 and 9 years. These ages are older than those in other parts of the world but consistent with reports of perinatally infected adolescents and young adults in sub-Saharan Africa [25, 26]. Programs for prevention of vertical HIV transmission and pediatric treatment in Botswana have been strengthened over the past decade and likely have resulted in earlier ART initiation among younger perinatally infected WLHIV [27].

We found that there were some differences in factors associated with HR-HPV by HIV infection status. Among women living with and without HIV, having ≥ 3 lifetime sex partners was associated with HR-HPV detection and this was statistically significant among those without HIV. Increasing number of lifetime sex partners is a well-documented risk factor for HPV infection, including in other studies of university students in sub-Saharan Africa [19]. Among women living without HIV, those who consistently used condoms in the past three months were significantly less likely to have HR-HPV detected compared to those who used condoms inconsistently or not at all. Although evidence about the protective effect of condom use on HPV infection in cross sectional studies is mixed, the finding in this report is consistent with findings from longitudinal studies on condom use [28, 29].

This study had some limitations. A convenience sample of university students and women attending HIV clinics in Gaborone, Botswana was obtained. Although these were recruited sequentially, the findings may not be fully generalizable to all females aged 18–22 years in Botswana. However, using similar recruitment methods in a subsequent study should produce a comparable population that can be used to assess vaccine impact. Next, the survey was administered by trained study staff, but social desirability bias could have impacted the reliability of self-reported sexual behavior risk factors. Further, because this study was not powered to evaluate risk factors for HPV detection, adjusted analyses should be interpreted with caution; we described some associations based on a higher than conventional p < 0.05 threshold and variables not selected in these models could possibly be associated with HR-HPV. Lastly, perinatal HIV infection status was self-reported and although status was verified in medical records if available, some participants may have been misclassified.

In conclusion, the findings from this study indicate a high prevalence of HPV, including HPV types targeted by the quadrivalent vaccine, in women living with and without HIV in Botswana. The Botswana HPV vaccination program is expected to have a substantial impact on reducing HPV infection and HPV-associated cancers. The data from this study will enable the first evaluation of HPV vaccine impact in Botswana in 3–4 years, when vaccinated girls reach the ages of 18–22 years. Importantly, the data will also allow future monitoring of HPV vaccine impact among WLHIV, including both perinatally and non-perinatally infected women, for which there is a critical gap of evidence. This report is a first step in understanding HPV vaccine impact in Botswana, which will inform and strengthen HPV vaccination programs in low- and middle-income countries worldwide.

## Data Availability

The data that support the findings of this study are available on request from the corresponding author. The data are not publicly available due to their containing information that could compromise the privacy of research participants.

## Abbreviations

HIV: Human immunodeficiency virus
HPV: Human papillomavirus
WLWH: Women living with HIV
HR-HPV: High-risk human papillomavirus
LR-HPV: Low-risk human papillomavirus
PR: Prevalence ratio
aPR: Adjusted prevalence ratio

## References

[1] Ramogola-Masire D. HPV vaccine for cervical cancer prevention in Botswana; 2016. http://www.commonwealthhealth.org/wp-content/uploads/2014/05/7-HPV-botswana-Masire.pdf.

[2] Forman D, de Martel C, Lacey CJ, Soerjomataram I, Lortet-Tieulent J, Bruni L (2012). Global burden of human papillomavirus and related diseases. Vaccine.

[3] de Martel C, Plummer M, Vignat J, Franceschi S (2017). Worldwide burden of cancer attributable to HPV by site, country and HPV type. Int J Cancer.

[4] Frisch M, Biggar RJ, Goedert JJ (2000). Human papillomavirus-associated cancers in patients with human immunodeficiency virus infection and acquired immunodeficiency syndrome. J Natl Cancer Inst.

[5] Joint United Nations programme on HIV/AIDS (UNAIDS). Botswana country page; 2016. http://www.unaids.org/en/regionscountries/countries/botswana. 12349391

[6] Botswana AIDS impact survey IV (BAIS IV); 2013. http://www.cso.gov.bw/images/aids_summary.pdf.

[7] Raesima MMFS, Voetsch AC (2015). Human papillomavirus vaccination coverage among school girls in a demonstration project—Botswana, 2013. MMWR Morb Mortal Wkly Rep.

[8] World Health Organization (2015). Human papillomavirus vaccines: WHO position paper, October 2014-recommendations. Vaccine.

[9] Binagwaho A, Wagner CM, Gatera M, Karema C, Nutt CT, Ngabo F (2012). Achieving high coverage in Rwanda’s national human papillomavirus vaccination programme. Bull World Health Organ.

[10] Drolet M, Benard E, Perez N, Brisson M, Group HPVVIS (2019). Population-level impact and herd effects following the introduction of human papillomavirus vaccination programmes: updated systematic review and meta-analysis. Lancet.

[11] Kahn JA, Xu J, Kapogiannis BG, Rudy B, Gonin R, Liu N (2013). Immunogenicity and safety of the human papillomavirus 6, 11, 16, 18 vaccine in HIV-infected young women. Clin Infect Dis.

[12] Giacomet V, Penagini F, Trabattoni D, Vigano A, Rainone V, Bernazzani G (2014). Safety and immunogenicity of a quadrivalent human papillomavirus vaccine in HIV-infected and HIV-negative adolescents and young adults. Vaccine.

[13] Looker KJ, Rönn MM, Brock PM, Brisson M, Drolet M, Mayaud P (2018). Evidence of synergistic relationships between HIV and human papillomavirus (HPV): systematic reviews and meta-analyses of longitudinal studies of HPV acquisition and clearance by HIV status, and of HIV acquisition by HPV status. J Int AIDS Soc.

[14] Okoye JO, Ofordile CA, Adeleke OK, Okechi O (2021). Prevalence of high-risk HPV genotypes in sub-Saharan Africa based on human immunodeficiency virus status: a 20-year systematic review. Epidemiol Health.

[15] Mbulawa ZZ, Coetzee D, Williamson AL (2015). Human papillomavirus prevalence in South African women and men according to age and human immunodeficiency virus status. BMC Infect Dis.

[16] McDonald AC, Tergas AI, Kuhn L, Denny L, Wright TC (2014). Distribution of human papillomavirus genotypes among HIV-positive and HIV-negative women in Cape Town, South Africa. Front Oncol.

[17] Singh DK, Anastos K, Hoover DR, Burk RD, Shi Q, Ngendahayo L (2009). Human papillomavirus infection and cervical cytology in HIV-infected and HIV-uninfected Rwandan women. J Infect Dis.

[18] Banura C, Mirembe FM, Katahoire AR, Namujju PB, Mbonye AK, Wabwire FM (2011). Epidemiology of HPV genotypes in Uganda and the role of the current preventive vaccines: a systematic review. Infect Agent Cancer.

[19] Bule YP, Silva J, Carrilho C, Campos C, Sousa H, Tavares A (2020). Human papillomavirus prevalence and distribution in self-collected samples from female university students in Maputo. Int J Gynaecol Obstet.

[20] Gilles C, Buljubasic M, Konopnicki D, Manigart Y, Barlow P, Rozenberg S (2020). Cervical, anal and oral human papillomavirus (HPV) infection in young women: a case control study between women with perinatally HIV infection and women with non-perinatally HIV infection. Eur J Obstet Gynecol Reprod Biol.

[21] Ananworanich J, Prasitsuebsai W, Kerr SJ, Hansudewechakul R, Teeratakulpisarn N, Saisawat K (2015). Cervical cytological abnormalities and HPV infection in perinatally HIV-infected adolescents. J Virus Erad.

[22] Kelly H, Weiss HA, Benavente Y, de Sanjose S, Mayaud P (2018). Association of antiretroviral therapy with high-risk human papillomavirus, cervical intraepithelial neoplasia, and invasive cervical cancer in women living with HIV: a systematic review and meta-analysis. Lancet HIV.

[23] Menon S, Rossi R, Zdraveska N, Kariisa M, Acharya SD, Vanden Broeck D (2017). Associations between highly active antiretroviral therapy and the presence of HPV, premalignant and malignant cervical lesions in sub-Saharan Africa, a systematic review: current evidence and directions for future research. BMJ Open.

[24] Bogale AL, Belay NB, Medhin G, Ali JH (2020). Molecular epidemiology of human papillomavirus among HIV infected women in developing countries: systematic review and meta-analysis. Virol J.

[25] Anderson K, Muloiwa R, Davies MA (2020). Long-term outcomes in perinatally HIV-infected adolescents and young adults on antiretroviral therapy: a review of South African and global literature. Afr J AIDS Res.

[26] Slogrove AL, Schomaker M, Davies MA, Williams P, Collaborative Initiative for Paediatric HIVE, Research Global Cohort C (2018). The epidemiology of adolescents living with perinatally acquired HIV: a cross-region global cohort analysis. PLoS Med.

[27] Keapoletswe K. Botswana PMTCT program.

[28] Winer RL, Hughes JP, Feng Q, O'Reilly S, Kiviat NB, Holmes KK (2006). Condom use and the risk of genital human papillomavirus infection in young women. N Engl J Med.

[29] Lam JU, Rebolj M, Dugue PA, Bonde J, von Euler-Chelpin M, Lynge E (2014). Condom use in prevention of human papillomavirus infections and cervical neoplasia: systematic review of longitudinal studies. J Med Screen.

